# Allele Interaction – Single Locus Genetics Meets Regulatory Biology

**DOI:** 10.1371/journal.pone.0009379

**Published:** 2010-02-23

**Authors:** Arne B. Gjuvsland, Erik Plahte, Tormod Ådnøy, Stig W. Omholt

**Affiliations:** 1 Centre for Integrative Genetics (CIGENE), Department of Mathematical Sciences and Technology, Norwegian University of Life Sciences, Ås, Norway; 2 Centre for Integrative Genetics (CIGENE), Department of Animal and Aquacultural Sciences, Norwegian University of Life Sciences, Ås, Norway; University of Chicago, United States of America

## Abstract

**Background:**

Since the dawn of genetics, additive and dominant gene action in diploids have been defined by comparison of heterozygote and homozygote phenotypes. However, these definitions provide little insight into the underlying intralocus allelic functional dependency and thus cannot serve directly as a mediator between genetics theory and regulatory biology, a link that is sorely needed.

**Methodology/Principal Findings:**

We provide such a link by distinguishing between positive, negative and zero allele interaction at the genotype level. First, these distinctions disclose that a biallelic locus can display 18 qualitatively different allele interaction sign motifs (triplets of +, – and 0). Second, we show that for a single locus, Mendelian dominance is not related to heterozygote allele interaction alone, but is actually a function of the degrees of allele interaction in all the three genotypes. Third, we demonstrate how the allele interaction in each genotype is directly quantifiable in gene regulatory models, and that there is a unique, one-to-one correspondence between the sign of autoregulatory feedback loops and the sign of the allele interactions.

**Conclusion/Significance:**

The concept of allele interaction refines single locus genetics substantially, and it provides a direct link between classical models of gene action and gene regulatory biology. Together with available empirical data, our results indicate that allele interaction can be exploited experimentally to identify and explain intricate intra- and inter-locus feedback relationships in eukaryotes.

## Introduction

Gregor Mendel's finding that hereditary units could be associated with particular observable traits and that in diploid organisms the apparent contribution from the two parents to a trait could be highly asymmetrical [1], led to the concepts additive and dominant (nonadditive) gene actions as well as the closely associated term *recessive* gene action. All three concepts have played a key role in the development of population genetics and quantitative genetics theory in evolutionary biology, production biology and biomedicine. Their presence or absence has substantial effects on key genetic features of populations like genetic variance, heritability, fixation rates of alleles, and long-term selection response.

The gene action associated with a biallelic locus is defined by the position of the genotypic value (i.e. the mean phenotypic value for individuals with a given genotype) of the heterozygote relative to the genotypic values of the two homozygotes [2], [3]. Thus, despite that much of current genetics theory is actually founded on *biallelic* single locus genetics, the basic gene action concepts used to construct the theory only depend on genotypes and their relative positions to each other. The emergence of molecular genetics did not change this situation. Notwithstanding the complex evolution of the gene concept in the molecular biology community [4], and that it has been known for a long time that the mRNA production from two alleles physically positioned on homologous chromosomes may be functionally dependent [5], the relational gene action concepts, based on an abstract gene notion, became part of the modern molecular genetics vocabulary without any semantic change. Even when parts of quantitative genetics was merged with molecular genetics into a methodology for identifying Quantitative Trait Loci (QTLs), one did not see the need for anchoring the gene action concept to the genotype level and thus come closer to mechanism. Subsequently, the whole body of current genetics theory, even when it deals with sequence and expression data, relies on gene action concepts that do not provide any information about the functional dependency between the two alleles composing each genotype.

Even though the classical gene action concepts still serve several purposes well, they cannot serve directly as mediators between genetics theory and regulatory biology. The establishment of such a link is sorely needed if we aim for a better understanding of the biological mechanisms underlying intra- and inter-locus additivity and nonadditivity. It would also be an important contribution to the development of a more systems oriented quantitative genetics theory based on how genes actually act and interact [6]–[13].

Here we show that by considering the additivity and nonadditivity features of each genotype explicitly, regulatory biology can be linked to the basic concepts of single locus genetic theory in a straightforward way. We start out by introducing the concept of *allele interaction*, which characterizes the degree of functional dependency between the two alleles composing each genotype of a given locus. We then demonstrate that the concept enriches single locus genetics with a number of new characterizing features, and show that it enforces the conclusion that genetic dominance is in fact given by a specific relationship between the allele interactions of all the three genotypes. Finally, we illustrate how the allele interaction concept succeeds in making the sorely needed link between current single locus gene action concepts and regulatory biology by use of gene regulatory models involving positive and negative feedback.

Our results suggest that the allele interaction concept can be used experimentally in a very direct way to identify and elucidate intricate intra- and inter-locus feedback relationships manifested at the mRNA and protein expression levels in eukaryotes. Even though this paper is conceptual in character, we emphasize already at this point that all premises underlying this work are either already well supported by empirical data or (together with the reported predictions) well within reach to be tested experimentally, and an experimental test program is indeed suggested.

## Results

### Definition of Allele Interaction

**Figure 1:**
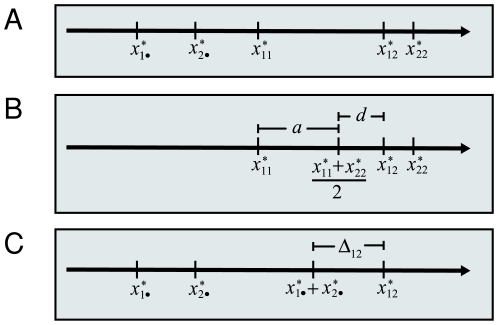
Example illustrating the classical definition of gene action and the proposed definition of allele interaction. (**A**) Phenotype axis with five genotypic values for five genotypes (two homozygote, one heterozygote and two hemizygotes) for a biallelic locus *X*. (**B**) The classical definition of gene action (eqn. (2)). Additive genotypic value 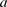 is defined as half the distance between the two homozygotes while dominance genotypic value 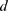 is defined as the difference between the heterozygote genotypic value and the midpoint between the two homozygote genotypic values. (**C**) Proposed definition of allele interaction (eqn. (1)). The allele interaction value 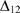 for the heterozygote is defined as the difference between the heterozygote genotypic value and the sum of the two hemizygote genotypic values.

Consider a biallelic locus *X* with homozygote genotypes 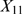 and 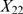, heterozygote 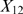 and the two hemizygotes (only one allele present) 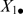 and 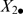. Presuming no environmental variation, we let 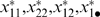 and 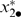 denote the corresponding genotypic values for a specific phenotypic trait, and let the allele indexes be such that 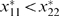 (see Fig. 1A for an illustration). We define the *allele interaction value* (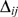) of genotype *X_ij_* as the difference between the biallelic genotypic value and the sum of the two hemizygote genotypic values. A biallelic locus is thus characterized by three such values (see also Fig. 1C):
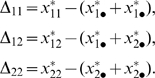
(1)


The locus shows *homoallelic nonadditivity* when 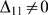 or 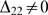 and *heteroallelic nonadditivity* when 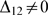. If 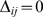, the biallelic genotype 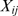 shows *additive allele action*, and *x_ij_* is simply the sum of the two hemizygotic genotypic values. As a combined effect is in general termed “additive” when it is the sum of the individual effects of its underlying components [14], the two alleles can then unambiguously be said to behave additively. Furthermore we say that the genotype *X_ij_* shows *positive allele interaction* if 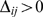 and *negative allele interaction* if 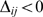. The three 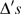 quantify the allelic functional dependencies underlying the genotype to phenotype mapping 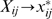. Our definitions of homoallelic and heteroallelic allele interactions provide a genotype level refinement of the “physiological” [15] and “functional” [16] descriptions of gene action, and can effortlessly be applied to already existing empirical data, see e.g. [17]–[21].

### Allele Interaction and Mendel's Genetic Dominance Concept

The classical additive and dominance genotypic values *a* and *d* of a biallelic locus are given by
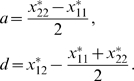
(2)(see [2] and Fig. 1B for an illustration). If *d* = 0, the locus is said to show additive gene action. The degree of dominance is often described by the scaled dominance value 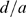. If 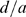 is positive, the heterozygote has a genotypic value larger than the mean of the two homozygotes, and the locus shows positive dominance (or positive nonadditive gene action). If 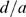 is negative, the heterozygote is positioned below the mean value, and the locus shows negative dominance (recessive, or negative nonadditive gene action). If the heterozygote has a genotypic value less than or greater than both homozygotes (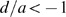 or 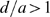), the locus shows negative or positive overdominance, respectively, with the term overdominance covering both cases (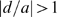).

By combining eqns. (1) and (2) it follows that *d* can be expressed solely in terms of the 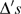:
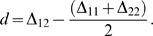
(3)


Thus, in contrast to a common conception among geneticists, *the value of d does not follow from whether the two alleles in the heterozygote act in an additive way or not, but is given by a specific relationship between the allele interaction values of all three biallelic genotypes*. For example, 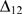 can be equal to zero and the locus may still show nonadditive gene action 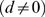, and 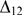 may be different from zero and the locus may display additive gene action 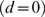.

The allele interaction concept enriches single locus genetics considerably as a biallelic locus may in principle display 18 (disregarding symmetrical situations) distinct *allele interaction sign motifs* (or *sign motifs* for brevity) 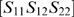, where 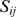 is the sign of 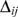 (Table 1, left column). For the first 11 sign motifs out of the total 18 cases, the sign of the dominance value (*d*) of the locus is uniquely determined. Among these 11 we observe that in the 6 cases where 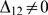, its sign is identical to the sign of the corresponding dominance value (3 negative, 3 positive). In the remaining 7 sign motifs, the sign of *d* depends not only on the signs of 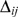, but also on their relative magnitudes (Table 1). This shows very clearly that the definition of genetic dominance cannot be used to infer allele interaction sign motifs, but the allele interaction concept can be used to infer dominance sign values. The allele interaction concept is thus unquestionably more fundamental than the dominance concept.

**Table 1:** Single locus allele interaction sign motifs and the sign of the corresponding genetic dominance *d*.

	Sign motif	Sign(*d*)
1	0 0 0	0
2	0 0 +	–
3	+ 0 +	–
4	+ – +	–
5	0 – 0	–
6	0 – +	–
7	0 + –	+
8	– 0 –	+
9	0 + 0	+
10	0 0 –	+
11	– + –	+
12	0 + +	–/0/+
13	0 – –	–/0/+
14	+ 0 –	–/0/+
15	+ + +	–/0/+
16	+ + –	–/0/+
17	+ – –	–/0/+
18	– – –	–/0/+

Because the allele interaction value 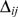 only depends on a single genotype, it is a measure of the functional dependency between the two alleles *i* and *j* in that particular genotype, and we will show in the next section that it indeed carries information about regulatory anatomy. Even though the above definitions can be consistently applied to any phenotypic feature in any diploid organism, the expression (mRNA or protein) phenotype defines a natural starting point from the point of view of data availability and regulatory complexity, and in the rest of the paper we will focus on the mRNA phenotype.

### Additive Allele Action Is a Prevailing Underlying Feature of Eukaryotic mRNA Levels

Several studies on yeast, mice and humans show that additive allele action (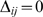) is a predominant phenomenon in eukaryotic gene expression systems. Two studies of the ratios of mRNA to DNA in chromosome segment copy-number mutants in yeast suggests that there is no global dosage-compensation mechanism [22], [23] and a study of common copy number variations in humans shows that copy number correlates strongly and linearly with expression level [24]. In accordance with this, Prescott *et al.* [25] found in a Df1 mouse model of the 22q11 deletion syndrome that none out of 21 genes adjacent to the transcription factor Tbx1 convincingly demonstrated a nonlinear response to Df1 hemizygosity at the mRNA level. Furthermore, intranuclear processing does not appear to be rate-limiting in general [26], such that the transcriptional initiation rate is also the exit rate of the mRNA to the cytoplasm [27].

Thus it seems fair to conclude that in most cases, trivial dosage or saturation effects associated with gene transcription process rates do not give rise to allele interaction at the mRNA expression level. This empirically observed robustness of the gene transcript production machinery to copy number variation opens for a systematic study of the relationship between 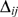 and gene regulatory mechanisms, using gene expression models that focus on regulatory aspects only.

Omholt *et al.* [12] introduced simple differential equation models for gene regulatory networks with one and two loci to better understand the regulatory conditions underlying dominance and additivity. In the following we show that by using the same methodological approach, but now with the allele interaction concept incorporated, we are able to systematically uncover relationships between gene regulatory mechanisms, allele interaction sign motifs and gene action. We start out by identifying the types of regulation that will be associated with the observed additive allele action reported above.

### Functional Independence Implies Additive Allele Action

Let *x*_1_ and *x*_2_ represent the concentration of mRNA produced by allele 1 and allele 2, respectively. The time rate of change of *x*_1_ and *x*_2_ can be modeled by
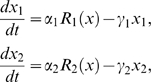
(4)where 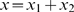 is the total mRNA concentration. The first term in the right-hand side of the equations represent the transcription rate, where 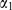 and 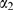 are the maximum transcription rates, and the dose-response functions *R*_1_ and *R*_2_ (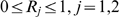) for the two alleles of the gene express how the transcription rate depends on the total mRNA concentration. The second term in the equations expresses that for each allele the degradation rate of the allele mRNA product is proportional to its own concentration, and 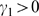 and 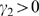 are the relative degradation rates [12].

There is usually a long chain of actions from the concentration of mRNA to the regulator that activates or inhibits the transcription of the gene. We might have considered the allele interactions of the phenotype defined by the equilibrium concentrations of any agent in this chain, but that would have required additional assumption on how this agent depends on the gene product concentration. Thus, considering the mRNA concentration allows us to use a simpler model with fewer assumptions about rate functions and conversion rates. In eqns. (4) the gene regulation is modeled *as if* the gene product acts directly as a transcription factor [28]. Interpreted in this way, the dose-response functions express the aggregated effect of the whole chain of actions from the initial mRNA transcription, its translation, protein folding, etc. to the final effect on the transcription rate. This kind of model has been widely studied and has been applied successfully to many gene regulatory systems (cf. [29]).

If the two eqns. (4) have identical parameters and rate functions, the system describes a homozygous locus, otherwise it describes a heterozygous locus. We will also consider the hemizygous locus case where either 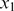 or 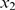 is identically zero because one of the alleles has been knocked out. We let the stable equilibrium concentration of the model variables represent the phenotype. The equilibrium values 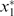 and 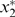 of the gene expression product concentration from the two alleles are found by solving the equations 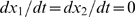 with respect to 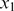 and 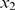, i.e.
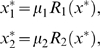
where 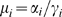 and 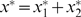. The three different genotypic equilibrium values 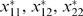 for the 11-homozygote, the 12-heterozygote and the 22-homozygote are then
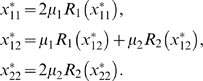
(5)


To compute the allele interaction values 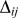 we also need the mono-allelic equilibrium values 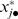, which are the equilibrium solutions of just one of eqns. (4) with 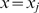, i.e.
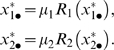
(6)


In Appendix S1 we show analytically for a generalization of eqns. (4) that a genotype shows lack of allele interaction 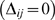 *if and only if* the dose-response functions 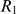 and 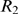 are constant, i.e. 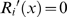 for both *i*. Thus according to this model framework, additive allele action for the expression level phenotype is the rule for genes where regulation of expression of one allele is independent of the expression level of the other allele (see Result 2 in Appendix S1). In more specific biological terms this situation implies that the gene is either constitutively expressed or under downstream regulatory control by one or more other genes. This leads to the prediction that genes showing the observed additive expression responses to copy number variation belong to these two categories. It also leads to the conclusion that *the appearance of allele interaction points to the presence of some specific regulatory mechanism that needs to be clarified*.

By assuming that additive allele action is the rule unless some particular regulatory situation causing allelic interdependency is involved, we are finally in position to theoretically address the relationship between gene regulatory mechanisms and allele interaction values different from zero. Even though empirical data suggest that the [0 0 0] sign motif is predominant, the empirical relevance and information contents of the remaining 17 allele interaction sign motifs (Table 1) need to be assessed. Below we start this assessment under the guidance of two pertinent questions: (i) how many of these 17 motifs can actually be realized by any given gene regulatory mechanism as a result of functional genetic variation at a single locus, and (ii) is any realized sign motif associated with specific regulatory mechanisms or not.

### Allele Interaction Sign Motifs for a Feedback-Regulated Locus

Negative and positive feedback are ubiquitous types of regulation in developing as well as adult organisms [27] and have been shown to be intimately connected both to the dominance concept [12], and to copy-number variation [5]. Feedback regulation therefore represents a natural starting point for elucidating the relationship between regulatory principles and the 17 possible sign motifs in the context of expression phenotypes. Negative and positive feedback are mathematically well-defined concepts when used in connection with ordinary differential equation systems, and the terms do not acquire different meanings in different contexts [5], [30].

In Appendix S1 we show analytically for a generalization of eqns. (4) that negative autoregulation generates a negative allele interaction sign and that positive autoregulation generates a positive allele interaction sign. This means that an autoregulatory feedback system can generate strictly additive behavior only when the feedback is not active around the steady state. In Appendix S1 we also derive a number of other analytic relationships, formulated as precise Results.

To obtain more quantitative insights concerning allele interaction values 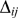, the distribution of the dominance values and the feedback regulation, we supplemented the analytical deductions with numerical experiments on eqns. (4). We explored three combinations of dose-response functions and genetic variation: (i) monotonic dose-response functions and noncoding variation only, (ii) monotonic dose-response functions and both noncoding and coding variation, and (iii) nonmonotonic dose-response functions and both noncoding and coding variation. Noncoding variation was modeled by varying maximal production rates and shape parameters for the dose-response functions, while coding variation was modeled by introducing positive weights 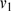 and 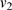 such that the response functions depend on 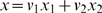. The weights reflect that the transcription factors coded by the two alleles differ in their binding affinity to the promoter.

#### Monotonic dose-response function and noncoding variation

We represented the monotonic dose-response functions by the common Hill function 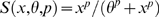, such that 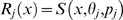 and 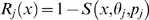 express positive and negative feedback, respectively. By assuming that the polymorphisms responsible for variation of the gene expression level were located in the *cis*-regulatory region only (i.e. in a position closely linked to the gene whose expression level variation they contribute to), their impact could be fully described by varying the parameters 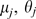, and 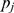 (see Methods for further details about the simulations).

**Figure 2:**
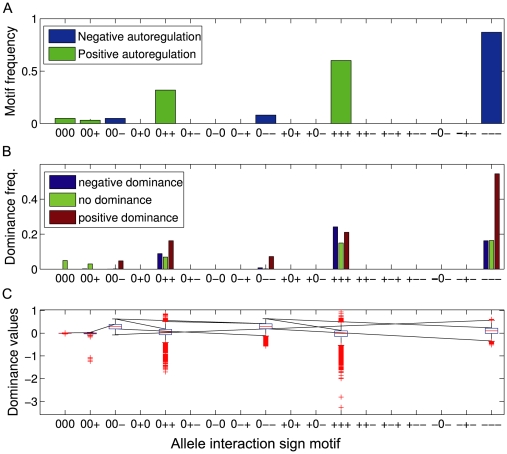
Allele interaction sign motifs and dominance generated by negative and positive autoregulation. (**A**) Relative frequency of the 18 different allele interaction sign motifs with positive and negative autoregulation. (**B**) The frequency distributions of negative dominance, no dominance, and positive dominance for the displayed sign motifs. (**C**) Box plots of the scaled dominance values (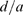) for the various sign motifs. See text for equations and parameter value ranges. 50000 simulations were run for each type of autoregulation, 47186 and 12054 valid datasets are shown for negative and positive autoregulation, respectively.

Negative autoregulation generated the sign motifs [0 0 –], [0 – –], and [– – –], where the first two appeared at low frequency (Fig. 2A). The sign motif [– – –] gave rise to mainly positive dominant gene action (Fig. 2B), but additive and negative dominant gene actions were also well represented. This shows that a locus may show additive gene action while being completely nonadditive at the allele interaction level. The reason why we encounter such a high frequency of additive gene action even in the [– – –] case is either that all the 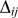 are very small, or the allele interaction value of the heterozygote almost equals the mean value for the two homozygotes. The three sign motifs characteristic of negative autoregulation generate mostly moderate partial dominance (Fig. 2C).

Positive autoregulation generated the allele interaction sign motifs [0 0 0], [0 0 +], [0 + +], and [+ + +] (Fig. 2A). The most frequent sign motif [+ + +] gave rise to all three types of gene actions (additive, positive dominance, negative dominance) in commensurable proportions (Fig. 2B). Additive gene action was also repeatedly generated by the second most frequent allele interaction sign motif [0 + +]. Even though [+ + +] and [0 + +] in most cases gave rise to moderate partial dominance (Fig. 2C), they were also prone to generate negative overdominance.

In summary, our model framework predicts that 7 out of the 18 allele interaction sign motifs can be generated by negative and positive autoregulation at reasonable frequencies when the polymorphisms are situated in the *cis*-regulatory region (negative: [0 0 –], [0 – –], [– – –]; positive: [0 0 0], [0 0 +], [0 + +], [+ + +]) (Fig. 2A). These 7 sign motifs define a distinct subclass among the 18 as they are characterized by the heterozygote having the same allele interaction sign as at least one of the homozygotes and that negative and positive allele interaction are not present in the same sign motif. Results 1–3 in Appendix S1 explain Figure 2A by showing that active negative (positive) autoregulation results in negative (positive) allele interaction, and that outside the region of active autoregulation there is additive allele action. The patterns in Figure 2C are also supported by Result 4 (Appendix S1) that negative autoregulation gives neither negative nor positive overdominance, and Result 5 (Appendix S1) that positive autoregulation (and 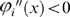, which is the case for the equilibrium points used in our simulations) open up for negative overdominance, but not positive overdominance.

#### Monotonic dose-response function and both noncoding and coding variation

Coding variation did not introduce any new sign motifs, but the sign motifs belonging to negative autoregulation generated negative as well as positive overdominance (see below for further discussion of overdominance).

#### Nonmonotonic dose-response function and both noncoding and coding variation

**Figure 3:**
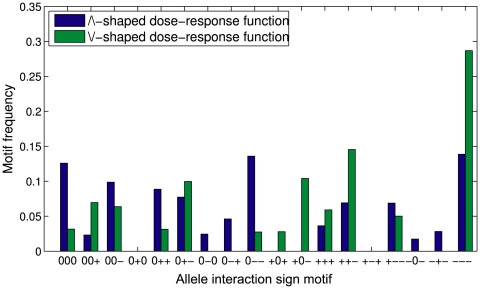
Allele interaction sign motifs generated by nonmonotonic autoregulation. Relative frequency of the 18 different sign motifs with feedback regulation and the nonmonotonic dose-response relationships in eqn. (7). 10000 simulations were run for each type of autoregulation, results for 9631 and 6527 valid datasets are shown for /\-shaped and \/-shaped dose-response functions, respectively (see Methods for parameter values and details).

The seven sign motifs characterized by containing at least one positive and one negative sign ([+ – +], [0 – +], [0 + –], [– + –], [+ 0 –], [+ + –], [+ – –]) (Table 1) define a second distinct subgroup. One way to achieve this type of sign motif in an autoregulatory regime is to let the gene regulatory function become nonmonotonic (Methods) such that the gene product is activating at low concentrations and inhibiting at high concentrations [31], [32], or *vice versa*. Such functions together with polymorphisms influencing production rates, decay rates or the weights 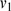 and 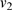 were able to generate 16 of the 18 sign motifs (Fig. 3). The two remaining ones, [+ – +] and [0 + 0], were also observed, but in less than 1% of the simulations. Thus, autoregulation is in principle capable of generating all allele interaction sign motifs that can be displayed by a biallelic locus.

#### Allele interaction sign motifs and higher-order feedback

Higher-order feedback systems bring up some additional aspects. We studied two cases of positive feedback and two cases of negative feedback between two loci among which only one was polymorphic, using the model presented in [12]. A positive two-element loop is composed of two negative actions or two positive actions. A negative two-element loop is composed of one negative and one positive action. In all four cases we monitored the gene expression from both the polymorphic and the nonpolymorphic locus. The results show very clearly the intimate relationship between intra- and inter-locus functional dependencies even under very simple conditions.

**Figure 4:**
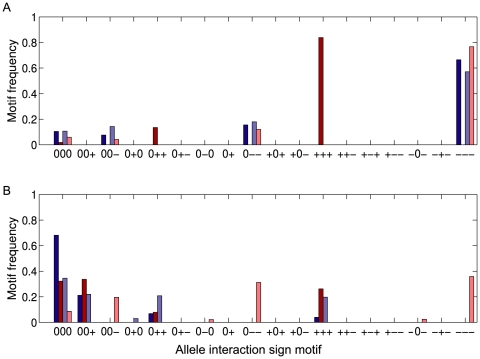
Allele interaction sign motifs generated by two-element feedback loops. Relative frequencies of allele interaction sign motifs displayed by four different two-element feedback loops when genetic variation is only present in locus 1. In both panels blue bars represent the steady state expression levels assayed at locus 1 (polymorphic), while red bars represent steady state expression levels assayed at locus 2 (nonpolymorphic). (**A**) Results for negative two-element loops. Dark colors: Locus 2 acts negatively on locus 1 (9454 valid datasets). Bright colors: Locus 2 acts positively on locus 1 (9346 valid datasets). (**B**) Results for positive two-element loops. Dark colors: Both loci act positively on each other (279 valid datasets). Bright colors: Both loci act negatively on each other (9411 valid datasets). Parameter ranges were the same as for the one-locus simulations. 10000 simulations were run for each type of two-element feedback loop.

In the negative feedback case where the polymorphic locus is activated by the other locus, the *cis*-variation creates the same allele interaction sign motifs as negative autoregulation except for the additional appearance of the [0 0 0] sign motif at a low frequency. The allele interaction sign motifs of the nonpolymorphic locus are the same as for the polymorphic one (Fig. 4A, dark colored bars). However, when the polymorphic locus is inhibited, the allele interaction sign motifs of the nonpolymorphic locus become the same as for positive autoregulation (Fig. 4A, bright colored bars).

In the positive feedback case two positive actions create the same sign motifs as for positive autoregulation for the polymorphic and the nonpolymorphic locus (Fig. 4B, dark colored bars), while two negative actions maintain the positive autoregulation sign motifs in the polymorphic locus and swap the sign motifs of the nonpolymorphic locus to those characterizing negative autoregulation (Fig. 4B, bright colored bars).

An exhaustive analytical and numerical analysis of the higher-order feedback cases will be presented elsewhere, but the patterns described for the two-element loop were conserved in simulations of a 3-element loop (data not shown). Higher-order feedback thus seems to generate the same allele action sign motifs in the polymorphic locus as autoregulation. This suggests that the allele interaction sign motifs characteristic of feedback may be generic.

### Allele Interaction Sign Motifs and Genetic Overdominance

We analyzed the single locus case in more detail to identify which of the realized allele interaction sign motifs were most prone to generate overdominance compared to partial or complete dominance. Negative autoregulation with monotonic dose-response and without coding variation generated no overdominance, in accordance with our analytical result (see Appendix S1, Result 4), while the inclusion of coding variation gave overdominance at the level of a few percent for the sign motifs [0 0 –], [0 – –] and [– – –]. Positive autoregulation with no coding variation generated about the same proportion of overdominance for the sign motifs [0 0 +], [0 + +] and [+ + +]. With coding variation the relative proportion of overdominance for the same sign motifs increases somewhat (but all <10%). However, in this latter situation a new sign motif is generated, [+ 0 +], and for this one the overdominance percentage is around 90%.

**Figure 5:**
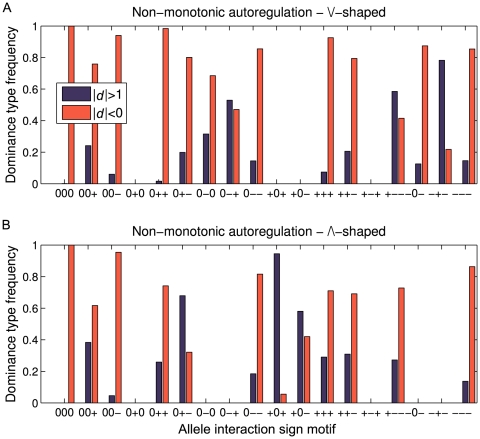
Overdominance generated by nonmonotonic autoregulation. Frequencies of partial dominance/additive gene action (red bars) and overdominance (blue bars) for autoregulation with nonmonotonic dose-response relationship. Frequencies of type of dominance are shown within each sign motif, see Fig. 2 for the corresponding overall sign motif frequencies. Results for /\-shaped and \/-shaped dose-response functions are shown in (**A**) and (**B**), respectively (see Methods for details).

The picture changes dramatically with nonmonotonic dose-response curves (Fig. 5). In this case 11 out of the 16 realized cases show substantial proportions of overdominance in one or both regulatory situations. Particularly the sign motifs [0 + –], [0 – +], [+ 0 +], [+ 0 –], [+ – –], and [– + –] stand out with overdominance percentages in the range 50–95%.

Due to the paucity of empirical data it is premature to elaborate much further on these new insights connecting the overdominance phenomenon to the allele interaction concept and feedback regulation. But considering the role overdominance is supposed to have in connection with generation of heterosis [33], [34], the results suggest that the allele interaction concept and the associated sign motifs may contribute to a better understanding of the heterosis phenomenon.

## Discussion

### Alternative Biological Mechanisms Leading to Allele Interaction

Several known biological mechanisms involving no regulatory feedback do indeed cause nonadditive allele interaction. These are for example transvection and sex-linked dosage compensation. Transvection-like phenomena have been described in a number of different organisms [35]. Most cases of transvection seem to involve nonreciprocal action of enhancers or silencers in *trans*. In these cases we predict that one will observe heteroallelic nonadditivity, but no homoallelic nonadditivity. This means that enhancer action in *trans* will generate the sign motif [0 + 0], while silencer action in *trans* will generate the sign motif [0 – 0], leading to positive and negative genetic dominance, respectively (Table 1). Further, dosage compensation is characterized by chromosome-wide adjustments of transcription. Female mammals deactivate one of their two X chromosomes in order to approximate the gene dosages of males, *C. elegans* hermaphrodites (XX) reduce transcription of each X by about one half, and *Drosophila* male flies increase transcription from their single X chromosome about twofold [36]. A biallelic locus showing dosage compensation will show the allele interaction sign motif [– – –]. There is reason to believe that there may be other sign motifs displayed by non-feedback mechanisms at the mRNA level than those listed above.

### Genetical Genomics and the Allele Interaction Concept

Quantitative trait locus (QTL) studies addressing the relationship between genetic variation and genome-wide mRNA expression levels in mouse and yeast, have shown that at least 25% of the so-called expression QTLs (eQTLs) are *cis*-acting QTLs [37]. *Our analysis suggests that any cis-acting eQTL showing genetic dominance points towards a possible feedback regulation or some other mechanism causing the nonadditivity*. Since homoallelic or heteroallelic nonadditivity is a prerequisite for genetic dominance (eqn. (3)), another prediction is that homozygous loci showing homoallelic nonadditivity are hotspots for the creation of genetic dominant eQTLs in the genome.

Although we included structural variation in allele products when investigating the relation between feedback and allele interaction sign motifs, our main focus was to elucidate the effects of regulatory variation. In general, regulatory variation is far less understood and studied than coding variation, but its importance for quantitative phenotypic variation and evolution through differential expression is highly significant [38]–[41]. Changes in expression levels have to be mediated by changes in either production or decay rates, and the literature contains several specific examples of noncoding mutations affecting for instance production rates [42], mRNA processing rates [43], [44], the shape of the cis-regulatory input function [45], [46], [47] and mRNA decay rates [48], [49], [50]. We think this makes a convincing case for considering variation in regulatory parameters as an expression of genetic variation.

### The Conceptual Connection between Allele Interaction and Previous Explanations of Genetic Dominance

Kacser and Burns proposed in 1981 an explanation for genetic dominance based on properties of metabolic systems [51]. They showed that dominance of the wild type over null alleles is an inevitable consequence of the kinetic properties of *n*-enzyme metabolic pathways when studied within the framework of metabolic control analysis. As this explanation has a very strong standing in the genetics community, we find it appropriate to make a few comments on how it relates to the current paper. We have previously shown that the K & B framework is quite restricted when it comes to explaining why recessive mutants are so common, the appearance of dominant mutations, the existence of functional recessive homozygotes, the phenomenon of overdominance, and how genetic dominance may arise from intralocus interaction [12]. This last feature is due to the fact that Kacser and Burns assumed that the enzymatic activity of the heterozygote is mid between the activity of the wild type homozygote and the null allele homozygote. Thus, the K & B explanation actually builds on the presumption that [0 0 0] is the default allele interaction sign motif for the involved enzymes. *This implies that it per definition cannot relate the nonadditivity between alleles in each genotype to the dominance value for RNA and protein phenotypes*.

### Experimental Program for How to Test Assumptions and Predictions

Our results open up for an extensive experimental research program on allele interaction in diploid organisms. Allele interaction across various environments can be surveyed in any diploid organism where gene knockouts can be produced. In particular, *S. cerevisiae* is very well suited as a model organism in this context since a collection of hemizygotes (i.e. heterozygous knockouts) for all nonessential genes is already available [52], [53]. In fact, all suggested experiments below could be done on budding yeast by use of available methodology and technology.

#### (i) Thoroughly test what available empirical data already suggest: genes with functionally independent alleles do not show allele interaction for expression level phenotypes (mRNA and protein) across a wide expression level range

The validity of this hypothesis can be established by measuring allele interaction for genes known to be downstream regulated or expressed from constitutive promoters. Use of heterologous constructions where one could adjust the promoter strength would provide the most conclusive data. A possible strategy would be to build upon a recently published protocol [54] which combines diverse loss-of-function alleles, to systematically modulate gene dosage in budding yeast. The use of this protocol on four enzyme-coding genes supports that [0 0 0] is a default sign motif [54].

#### (ii) Test our predictions about the relation between feedback regulation and allele interaction sign motifs

This can be done by inserting heterologous feedback loops with and without coding variation and with monotonic and nonmonotonic dose-response curves in diploid eukaryotic cells.

#### (iii) Determine allele interaction values and allele interaction sign motifs at the mRNA or protein level on a genome-wide scale across a wide range of environments

Given that the default sign motif is [0 0 0], this is likely to provide new information about gene regulation in eukaryotes. Moreover, allele interaction may be caused by quite complex feedback mechanisms involving much more than the direct interactions associated with binding of transcription factors to promoters or protein-protein interactions. This suggests that the allele interaction concept can be used to search for subtle feedback loops (including for instance metabolite levels) not easily detected by network analyses and bioinformatics methods.

#### (iv) Trace allele interaction through a hierarchy of phenotypes

The clear correspondence between feedback and allele interaction sign motifs is likely to become much more blurred when phenotyping at the metabolic, morphological, physiological or whole organism level. However, insights into the sign and strength of allele interaction arising at various phenotypic levels obtained by causally cohesive genotype-phenotype models [55] in combination with high-dimensional phenotyping is likely to provide important input to the long-standing discussion on the nature, origin and importance of nonadditive gene action.

### Concluding Remarks

Our theoretical analysis clearly demonstrates that single locus genetics is more complex than currently envisaged. The classical concepts of dominant or additive gene actions do not follow from whether the monoallelic contributions of the two different alleles in the heterozygote act in an additive way or not, but are given by a specific relationship between the degree of allele interaction for all the three genotypes. The concepts homo- and heteroallelic allele interactions open for a systematic investigation of the relation between allele interaction sign motifs and gene regulatory mechanisms. The elucidation of one of the oldest concepts in genetics appears to lead to a new experimental approach on how to identify intricate intra- and inter-locus feedback relationships in eukaryotes as well as to provide a most needed directly operational conceptual link between genetics theory and regulatory biology.

## Methods

### Monotonic Dose-Response Function and Noncoding Variation Only

In all simulations the 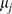 were sampled randomly from 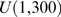 (the uniform distribution over (1,300)). The thresholds 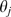 were sampled randomly from 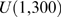, while the steepness parameters 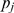 were sampled randomly from 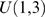. Since the equations can easily be rescaled [56], the actual parameter ranges are not critical, but by varying both 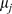 and thresholds we ensure that the simulations cover every regulatory situation from genes being permanently switched off to constitutively on. For each parameter set a random initial condition was chosen, and the biallelic and monoallelic equations were integrated numerically until a stable state was reached. In order to avoid null alleles, datasets in which one of the homozygotes gave a steady state level <0.01 were discarded. The value of 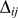 was set equal to zero if the sum of the monoallelic phenotypes differed less than 5% from the biallelic phenotype. Likewise, the dominance value *d* was set equal to zero if the heterozygote differed less than 5% from the mean of the homozygotic values. In order to avoid numerical artifacts and to obtain robust predictions, all allele interaction sign motifs with a frequency <1% were discarded.

### Monotonic Dose-Response Function and Both Noncoding and Coding Variation

We modeled coding variation by introducing weights 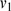 and 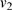 in 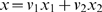. The weights were sampled from 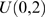.

### Nonmonotonic Dose-Response Function and Both Noncoding and Coding Variation

To study a nonmonotonic dose-response situation we used the response functions 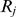 and 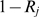, where
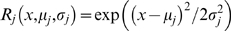
(7)is the probability density function of the normal distribution scaled such that the maximum function value is 1. Thus 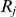 and 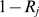 are nonmonotonic with /\-shape and \/-shape, respectively. We ran simulations as described above. The values of 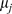 and 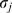 were sampled from 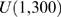 and 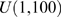, respectively.

## Supporting Information

Appendix S1 This appendix contains an analytic treatment of a generalization of the gene regulatory model given in eqs. (4)–(6). We present results on stability, allele interaction and genetic dominance.
(0.07 MB PDF)
