# Supplementary material for: Allele Interaction – Single Locus Genetics Meets Regulatory Biology
Source: PLoS One. 2010 Feb 23;5(2):e9379. doi: 10.1371/journal.pone.0009379 (PMC2826424; doi:10.1371/journal.pone.0009379)
Supplement: Appendix S1 — This appendix contains an analytic treatment of a generalization of the gene regulatory model given in eqs. (4)–(6). We present results on stability, allele interaction and genetic dominance. (0.07 MB PDF) [file pone.0009379.s001.pdf]

Appendix S1

Here we present a more detailed theoretical analysis of the gene regulatory model given in eqns. (4)-(6) and its equilibrium properties. However, we study the more general autoregulatory single-locus system

$$\dot{x}_1 = f_1(x, x_1), \qquad \dot{x}_2 = f_2(x, x_2), \tag{A.1}$$

where the rate functions f_1 and f_2 are continuous and differentiable with respect to their arguments and their parameters, and the dot denotes the derivative with respect to *t*. This form is more convenient than the equivalent (and more familiar) form $\dot{x}_i = g_i(x_1, x_2)$ because we want to analyse the stable states of system (A.1) in the same way as eqns. (4). We will establish the connection between the sign of the feedback loop and the sign of the allele interaction as defined in and after eqn. (1) as well as other relations between allele interaction values, equilibrium values and the value of *d*. As in the main text we consider the genotypic equilibrium values $x_{11}^*, x_{12}^*, x_{22}^*$ for the homozygotes and the heterozygote, and $x_{j\bullet}^*$ for the hemizygotes. When the locus is regulated by positive feedback, there could be multistationarity, and we assume there is only one stable point. If there were multistationarity, the equilibrium values would not only depend on allelic composition and parameter values, but also on initial conditions, and a more detailed and specific analysis would be necessary. Without loss of generality we index the alleles such that $x_{11}^* \le x_{22}^*$.We assume that all steady points are asymptotically stable, that unless otherwise stated $\partial f_i / \partial x$ and $\partial f_i / \partial x_i$ have fixed signs in a domain *D* containing all the stable points, and that

$$\frac{\partial f_1}{\partial x_1}\frac{\partial f_2}{\partial x_2} > 0, \quad \frac{\partial f_1}{\partial x}\frac{\partial f_2}{\partial x} \ge 0$$

everywhere in *D*. The well-known implicit function theorem allows us to consider the stationarity conditions $\dot{x}_1 = \dot{x}_2 = 0$ as equations that define x_1 and x_2 as functions of *x*, i.e. $x_i = \varphi_i(x)$. The five genotypic equlibrium values are given as the solutions of

$$
\begin{aligned}
x_{11}^* &= 2\varphi_1(x_{11}^*),\\
x_{12}^* &= \varphi_1(x_{12}^*) + \varphi_2(x_{12}^*),\\
x_{22}^* &= 2\varphi_2(x_{22}^*),\\
x_{1\bullet}^* &= \varphi_1(x_{1\bullet}^*),\\
x_{2\bullet}^* &= \varphi_2(x_{2\bullet}^*).
\end{aligned} \tag{A.2}
$$

The derivatives of $\varphi_i(x)$ are given by

$$
\frac{\partial f_i}{\partial x} + \frac{\partial f_i}{\partial x_i}\varphi_i{}'(x) = 0. \tag{A.3}
$$

This implies that the functions $\varphi_i(x)$ are well-defined and differentiable in D and monotonically increasing or decreasing. All the results in this appendix can easily be applied to the dose-response functions $R_i(x)$ in eqn. (4) because in this case, $\varphi_i(x) = \mu_i R_i(x)$.

Stability analysis

Mono-allelic (hemizygote) equilibrium points. For the equilibrium points defined by eqn. (6) the stability assumption implies

$$
\left.\frac{\partial f_i}{\partial x}\right|_{x=x_{i\bullet}^*} + \left.\frac{\partial f_i}{\partial x_i}\right|_{x_i=x_{i\bullet}^*} < 0.
$$

Inserting eqn. (A.3) we get

$$
\frac{\partial f_i}{\partial x_i}[1-\varphi'_i(x)]_{x=x_{i\bullet}^*} < 0. \tag{A.4}
$$

Biallelic equilibrium points. The Jacobian of the system (A.1) is

$$
J = \begin{bmatrix} \frac{\partial f_1}{\partial x} + \frac{\partial f_1}{\partial x_1} & \frac{\partial f_1}{\partial x} \\ \frac{\partial f_2}{\partial x} & \frac{\partial f_2}{\partial x} + \frac{\partial f_2}{\partial x_2} \end{bmatrix}.
$$

Stability of x_{ij}^* implies

$$
\begin{aligned}
\left.\det(J)\right|_{x=x_{ij}^*} &= \frac{\partial f_i}{\partial x}\frac{\partial f_j}{\partial x_j} + \frac{\partial f_i}{\partial x_i}\frac{\partial f_j}{\partial x} + \frac{\partial f_i}{\partial x_i}\frac{\partial f_j}{\partial x_j}\\
&= \frac{\partial f_i}{\partial x_i}\frac{\partial f_j}{\partial x_j}[1-\varphi_i{}'(x_{ij}^*) - \varphi_j{}'(x_{ij}^*)] > 0.
\end{aligned}
$$

This gives the three stability conditions

$$1-2\varphi_1'(x_{11}^*)>0,$$
$$1-\varphi_1'(x_{12}^*)-\varphi_2'(x_{12}^*)>0, \qquad \text{(A.5)}$$
$$1-2\varphi_2'(x_{22}^*)>0,$$

for the three biallelic genotypes.

Allele interaction in single locus regulatory models

***Result* 1.** If $\varphi_i'(x)<0$, then $\Delta_{ij}\le 0$, and if $\varphi_i'(x)>0$, then $\Delta_{ij}\ge 0$.

To prove this we first consider the case $\varphi_i'(x)<0$. Assume $x_{12}^*<x_{1\bullet}^*$. Then $\varphi_1(x_{1\bullet}^*)+\varphi_2(x_{1\bullet}^*)<\varphi_1(x_{12}^*)+\varphi_2(x_{12}^*)=x_{12}^*$, i.e. $x_{1\bullet}^*+\varphi_2(x_{1\bullet}^*)<x_{12}^*$, which contradicts the assumption. Thus, $x_{1\bullet}^*\le x_{12}^*$ and similarly, $x_{2\bullet}^*\le x_{12}^*$, which also are obvious from a graphical illustration. This implies

$$x_{12}^*=\varphi_1(x_{12}^*)+\varphi_2(x_{12}^*)\le\varphi_1(x_{1\bullet}^*)+\varphi_2(x_{2\bullet}^*)=x_{1\bullet}^*+x_{2\bullet}^*,$$

showing that $\Delta_{12}\le 0$. In the same way we can show that $\Delta_{11}\le 0$ and $\Delta_{22}\le 0$.

Then consider the case $0<\varphi_i'(x)<1$. In fact the stability conditions (A.5) put stronger restrictions on the derivatives, but here the present conditions are sufficient. Again assume $x_{12}^*<x_{1\bullet}^*$. Then

$$\varphi_1(x_{1\bullet}^*)+\varphi_2(x_{1\bullet}^*)-\left(\varphi_1(x_{12}^*)+\varphi_2(x_{12}^*)\right)<x_{1\bullet}^*-x_{12}^*,$$

which leads to the false result $\varphi_2(x_{1\bullet}^*)<0$. Thus, $x_{1\bullet}^*\le x_{12}^*$ and similarly, $x_{2\bullet}^*\le x_{12}^*$. This implies

$$x_{12}^*=\varphi_1(x_{12}^*)+\varphi_2(x_{12}^*)\ge\varphi_1(x_{1\bullet}^*)+\varphi_2(x_{2\bullet}^*)=x_{1\bullet}^*+x_{2\bullet}^*,$$

showing that $\Delta_{12}\ge 0$. In the same way we can show that $\Delta_{11}\ge 0$ and $\Delta_{22}\ge 0$.

Next we derive a formula for the allele interaction values (1) for the gene described by eqns. (3). Applying the mean value theorem we get

$$\begin{aligned}\Delta_{12}&=x_{12}^*-x_{1\bullet}^*-x_{2\bullet}^*\\&=\varphi_1(x_{12}^*)+\varphi_2(x_{12}^*)-x_{1\bullet}^*-x_{2\bullet}^*\\&=\varphi_1(x_{1\bullet}^*)+\varphi_1'(c_{12})[x_{12}^*-x_{1\bullet}^*]+\varphi_2(x_{2\bullet}^*)+\varphi_2'(c_{21})[x_{12}^*-x_{2\bullet}^*]-x_{1\bullet}^*-x_{2\bullet}^*\\&=\varphi_1'(c_{12})[x_{12}^*-x_{1\bullet}^*]+\varphi_2'(c_{21})[x_{12}^*-x_{2\bullet}^*],\end{aligned}$$

where $c_{12}\in<x_{1\bullet}^*,x_{12}^*>$, $c_{21}\in<x_{2\bullet}^*,x_{12}^*>$. Rearranging the top equality and inserting into the bottom one we arrive at

$$\Delta_{12} = \frac{\varphi_1'(c_{12})x_{2\bullet}^* + \varphi_2'(c_{21})x_{1\bullet}^*}{1-\varphi_1'(c_{12})-\varphi_2'(c_{21})}. \qquad \text{(A.6)}$$

In a similar fashion the allele interaction values for the two homozygotes can be expressed as

$$\begin{aligned}\Delta_{11} &= \frac{2\varphi_1'(c_{11})x_{1\bullet}^*}{1-2\varphi_1'(c_{11})}, \quad c_{11} \in < x_{1\bullet}^*, x_{11}^* >,\\ \Delta_{22} &= \frac{2\varphi_2'(c_{22})x_{2\bullet}^*}{1-2\varphi_2'(c_{22})}, \quad c_{22} \in < x_{2\bullet}^*, x_{22}^* >.\end{aligned} \qquad \text{(A.7)}$$

Because we already know that Δ_{ij} has the same sign as $\varphi_i'(x)$, the denominators in these three expressions are necessarily positive.

By inserting the expression for $\varphi_i'(x)$ obtained from eqn. (A.3) into (A.6) and (A.7) we find that $\Delta_{ij} = 0$ if and only if $\partial f_i / \partial x = \partial f_j / \partial x = 0$, This implies

***Result* 2.** *Additive allele action* for the gene described by eqns. (A.1) is observed if and only if the rate of change of expression of one allele is independent of the level of expression of the other allele.

Combining eqn. (A.7) with the definitions of Δ_{ii} in eqns. (2) we arrive at

$$\Delta_{ii} = \frac{\varphi_i'(c_{ii})}{1-\varphi_i'(c_{ii})}x_{ii}^*, \qquad \text{(A.8)}$$

and

$$x_{i\bullet}^* = \frac{1-2\varphi_i'(c_{ii})}{2(1-\varphi_i'(c_{ii}))}x_{ii}^*. \qquad \text{(A.9)}$$

We know from eqn. (A.7) that the numerator in eqn. (A.9) is positive, and since the dynamic system (A.1) is a positive system, the denominator is positive as well. We now apply the above results to the model eqn. (A.1) to get

***Result* 3.** All three genotypes of a gene *X* obeying eqn. (A.1) and which is under strict negative (positive) autoregulation will show negative (positive) allele interaction for their steady state expression levels.

By assuming strict negative or positive autoregulation we exclude the case of constant dose-response functions. This means that non-zero allele interaction will only be

observed as long as the gene is under active feedback regulation. If a genetic variant moves the steady state value into a region where the dose-response function is flat, then there is no active autoregulation and additive allele action.

Dominance in single locus regulatory models

Starting with the classical definitions $a = \left(x_{22}^* - x_{11}^*\right)/2$ and $d = x_{12}^* - \left(x_{11}^* + x_{22}^*\right)/2$ and proceeding as above, systematically expressing x_{12}^* first as $\varphi_1(x_{12}^*) + \varphi_2(x_{12}^*)$ and then in terms of *d* and x_{ii}^*, we find

$$\begin{aligned} d &= \frac{\varphi_1'(b_{11}) - \varphi_2'(b_{22})}{1 - \varphi_1'(b_{11}) - \varphi_2'(b_{22})} \frac{x_{22}^* - x_{11}^*}{2} \\ &= \frac{\varphi_1'(b_{11}) - \varphi_2'(b_{22})}{1 - \varphi_1'(b_{11}) - \varphi_2'(b_{22})} a, \end{aligned} \tag{A.10}$$

where $b_{ii} \in < x_{ii}^*, x_{12}^* >$, *i* = 1,2. This formula seems to express *d* only in terms of homozygote values, but *d* depends on x_{12}^* indirectly through b_{ii}. Note that $b_{ii} \neq c_{ii}$ as defined above. We let *N* represent the denominator in eqn. (A.10).

We next express eqn. (A.10) as $d = ra$ and use it to investigate the possibility of positive overdominance, i.e. $d/a = r > 1$ or $x_{12}^* > x_{22}^*$. We first consider the case of negative derivatives (negative autoregulation), in which it is trivial that *N* is positive. Assume there is positive overdominance. Then

$$r - 1 = \frac{2\varphi_1'(b_{11}) - 1}{1 - \varphi_1'(b_{11}) - \varphi_2'(b_{22})} > 0,$$

implying $\varphi_1'(b_{11}) > 1/2$, contrary to our assumption. In the same way we can show that the assumption of negative overdominance ($d/a = r < -1$ or $x_{12}^* < x_{11}^*$) also leads to a contradiction. We have then shown

***Result* 4.** For negative feedback (negative derivatives) the system (A.1) never shows overdominance.

In the case of positive derivatives we can draw definite conclusions when $\varphi_i(x)$ is two times differentiable with fixed sign for the second order derivative. We first consider $\varphi_i'(x) > 0, \varphi_i''(x) < 0$ which we deem the most likely situation in practice.

Again assume there is positive overdominance. Then $x_{ii}^* < b_{ii} < x_{12}^*$ for both *i*, and $N > 1 - \varphi_1'(x_{11}^*) - \varphi_2'(x_{22}^*)$ which is positive due to eqn. (A.5). Proceeding as in the proof of Result 4, we show that there can be no positive overdominance. Then assume there is negative overdominance. In this case $x_{12}^* < b_{ii} < x_{ii}^*$. Again this implies $N > 0$, and $\varphi_i'(b_{22}) > 1/2$. Even though $\varphi_i'(x_{22}^*) < 1/2$ due to eqn. (A.5), this is possible because $\varphi_2'(b_{22}) > \varphi_2'(x_{22}^*)$. In the same way we can show that if $\varphi_i'(x) > 0, \varphi_i''(x) > 0$, there can be positive overdominance but no negative overdominance. In summary

***Result* 5.** For $\varphi_i'(x) > 0, \varphi_i''(x) < 0$ there can be negative overdominance, but no positive overdominance in the system (A.1). For $\varphi_i'(x) > 0, \varphi_i''(x) > 0$ the system may show positive overdominance, but no negative overdominance.

In all the cases studied above, the denominator N in eqn. (A.10) is positive. Even though $N < 0$ cannot be excluded in general, $N > 0$ seems to be the most likely case, always fulfilled for negative derivatives and in many cases for positive derivatives. In these cases, $\text{sign}(d) = \text{sign}\left(\varphi_1'(b_{11}) - \varphi_2'(b_{22})\right)$. For negative feedback this is equivalent to $\text{sign}(d) = \text{sign}\left(|\varphi_2'(b_{22})| - |\varphi_1'(b_{11})|\right)$. In both cases the allele with the steepest $\varphi_i(x)$ is the dominant one. This gives our final result

***Result* 6.** Assume there is negative feedback in the system (A.1) and that the value of $|\varphi_i'(x)|$ is consistently larger for one allele than for the other. Then the dominant allele is the one with the steepest curve $y = \varphi_i(x)$.
